# Thermodynamics driving phytochemical self-assembly morphological change and efficacy enhancement originated from single and co-decoction of traditional chinese medicine

**DOI:** 10.1186/s12951-022-01734-w

**Published:** 2022-12-12

**Authors:** Xuemei Huang, Xiaojing Liu, Xiaoyu Lin, Zhihua Yuan, Yaozhi Zhang, Zhijia Wang, Wenmin Pi, Haoqiang Zhao, Haimin Lei, Penglong Wang

**Affiliations:** School of Chinese Pharmacy, Beijing University of Chinese Medicine, Beijing, 102488 China

**Keywords:** Traditional Chinese medicine, Self-assembly, Thermodynamics, Morphological chang e, Antibacterial enhancement

## Abstract

Through the self-assembled strategy to improve the clinical efficacy of the existing drugs is the focus of current research. Herbal formula granule is a kind of modern dosage form of traditional Chinese medicine (TCM) which has sprung up in recent decades. However, whether it is equivalent to the TCM decoction that has been used for thousands of years has always been a controversial issue. In this paper, taking the herb pair of *Coptidis Rhizoma*—*Scutellariae Radix* and its main component berberine—baicalin as examples, the differences and mechanisms of self-assemblies originated from the co-decoction and physical mixture were studied, respectively. Moreover, the relationship between the morphology and antibacterial effects of self-assemblies was illuminated via multi-technology. Our study revealed that the physical mixture’s morphology of both the herb pair and the phytochemicals was nanofibers (NFs), while their co-decoction’s morphology was nanospheres (NPs). We also found that the antibacterial activity was enhanced with the change of self-assemblies’ morphology after the driving by thermal energy. This might be attributed to that NPs could influence amino acid biosynthesis and metabolism in bacteria. Current study provides a basis that co-decoction maybe beneficial to enhance activity and reasonable use of herbal formula granule in clinic.

**Supplementary Information:**

The online version contains supplementary material available at 10.1186/s12951-022-01734-w.

## Introduction

In recent years, the research of small molecular phytochemical drugs has entered a bottleneck stage because of their poor selectivity, low bioavailability and other unexpected flaws [1]. Meanwhile, improving the clinical application of existing drugs by self-assembly has become one of the hot topics [2]. This kind of carried-free self-assemblies achieve drug repurposing, improve agent activity without creating new risks [3, 4]. Self-assembly refers to molecules assembled into supramolecular structures by the action of non-covalent bonds (hydrogen bonding, coordination bonding, electrostatic attraction or π-π stacking). For instance, rhein, a star molecule originated from herb medicine, could be directly self-assembled to form sustained-release gels through non-covalent bonds such as π-π stacking, which could significantly enhance its anti-neuroinflammatory activity [3]. In addition, several clinical nucleoside anti-tumor drugs could self-assemble into stable nanoparticles with few side effects and excellent efficacy [4]. Recently, our previous studies also found that the small molecule natural products could enhance activity or reduce toxicity after self-assembly. For example, berberine (BBR) based self-assemblies could form stable and carried-free NPs with good biocompatibility, which would adhere to the surface of bacteria and increase the drug concentration in the local environment [5, 6]. In contrast, BBR and aristolochic acid could block the toxic groups of aristolochic acid through self-assembly to form linear multiphase supramolecules, thus reducing the acute nephrotoxicity of aristolochic acid [7]. Based on these studies, self-assembly strategy could be considered as a new idea to improve clinical efficacy and modify toxicity of herb medicine.

For thousands of years, Chinese herbal medicine has played a vital role in the prevention and treatment of diseases; and it has formed a unique system of traditional Chinese medicine (TCM), which is an inseparable part of Chinese traditional culture [8]. As one of the new forms of TCM prescriptions, herbal formula granule has been widely used because of its easy transportation, simple way of taking and controllable quality [9, 10]. It not only has been popularly in China, but also used in Asian countries such as South Korea and Japan, and gradually admitted by the pharmaceutical markets of western countries such as Britain, United States, Germany and so on [11]. In China, more than 700 kinds of herbal formula granules has been applied in clinical use, and the annual market sales are billions of dollars [12, 13]. However, since the modern formulation granule came into being, compared with the thousands of years’ traditional decoction, it had been facing an important controversial focus due to herbal formula granules lacking the process of co-decocting the combination herb medicines. Previous studies have shown that many phytochemical components undergo variously physical and chemical changes in the process of decocting [14, 15], which could affect the clinical efficacy. For example, there are nanoparticles with the size about 100 nm in the Baihu decoction with complex composition, which has a good antipyretic effect on the rabbit fever model induced by lipopolysaccharide [16]. There were also NPs with different sizes in the Gegenqinlian decoction, and the main components of the NPs were puerarin, baicalin, berberine and so on [17]. All these results indicated that the decocting process could produce the self-assemblies and affect the biological activity of herb drugs in clinic; and self-assembly strategy might be helpful to enhance clinical efficacy of formula granules.

*Coptidis Rhizoma* (*Coptis chinensis Franch*, CR) and *Scutellariae Radix* (*Scutellaria baicalensis Georgi*, SR) are classical TCM combinations in clinic, which originated from *Treatise on Febrile Diseases (Shang Han Lun)* in 219 AD. As the herb pair, CR-SR are used together to mutually reinforce efficacy, such as Huanglian jiedu decoction, Gegenqinlian decoction and Xiexin decoction and the like [18–20]. Currently, many studies have shown there were phytochemical self-assembly to form supramolecule in the process of co-decoction of CR-SR [21–23]. Our previous studies had clearly exploited that BBR and baicalin (BA), both the main components of CR-SR herb pair, could interact to form spherical NPs during co-decoction; and compared with BBR, the inhibition ability of self-assembly against *Staphylococcus aureus* (*S.aureus*) was significantly improved [24]. Similar results had also been proved in other phytochemical self-assemblies from TCM decoctions [25]. Therefore, co-decocting of herb medicine is one of the necessary conditions to form the phytochemical self-assemblies, which can influence the clinical effect. While the mechanism of co-decocting thermodynamic process in the influence on the activity of TCM self-assembly is still unclear. Simultaneously, as the self-assemblies respond to external stimuli (such as solvent type, pH value or temperature), the stacking arrangement of assembly units would change, resulting in morphological transitions [26–30].

**Fig. 1:**
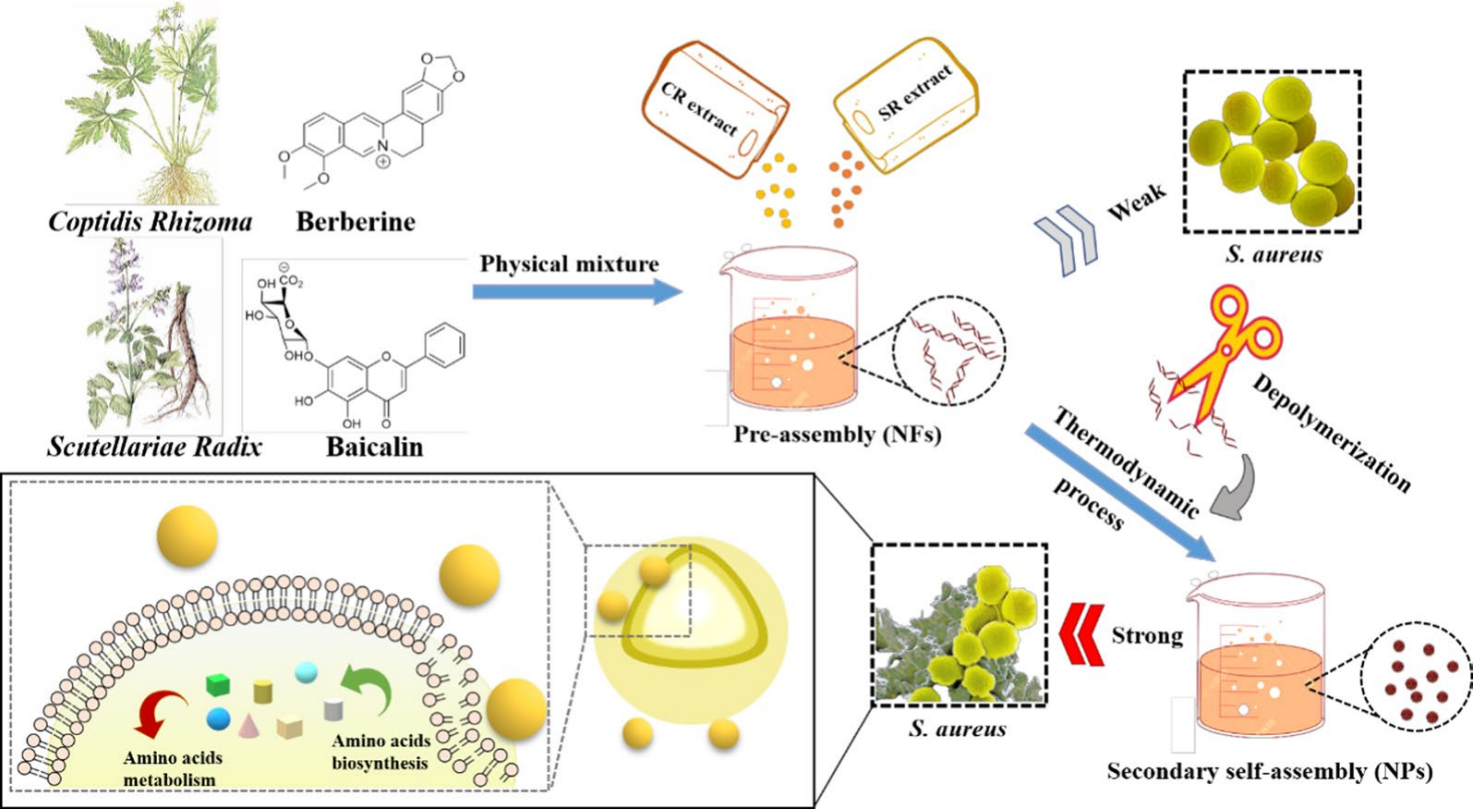
The differences of self-assemblies formed by physical mixing and decocting

Based on the classical Chinese herb medicine pair of CR/SR and its main components, we observed the morphological characteristics of the NPs and NFs formed by co-decoction and physical mixture, respectively. Using multi spectroscopic technologies, the self-assemblies’ differences in spatial configuration between the NPs and NFs were elucidated. Moreover, the relationship between the two kinds of self-assemblies and biological activity was further discussed on *S.aureus* model. Morphological observation of bacteria and RNA-seq test revealed that the active enhancement of NPs was attributed to its affection on cell surface, then further influenced amino acid biosynthesis and metabolism (Fig. 1). These data showed the co-decoction might be beneficial to improve the antibacterial effect and provided a reliable scientific basis to improve herbal formula granules’ clinical efficacy.

## Materials and methods

### Materials

*Scutellariae Radix* (CR) and *Coptidis Rhizoma* (SR) were all purchased from Tongrentang in Beijing. Berberine hydrochloride (BBR, C_20_H_18_ClNO_4_, 98%), Baicalin (BA, C_21_H_18_O_11_, 98%) and crystal violet (≥ 10 mM, 1 mL in DMSO) were purchased from Aladdin (China). Acetonitrile was purchased from Fisher (USA). Phosphoric acid was purchased from Sigma-Aldrich (USA). PBS buffer solution was purchased from Wuhan Sevile Biotechnology Co., Ltd (China). Nutrient broth (NB) and nutrient agar were purchased from Beijing Aboxing Biotechnology Co., LTD (China). Glucose and acetic acid were purchased from Beijing Chemical Plant (China).

### Preparation of TCM decoctions

Firstly, CR or SR was weighed and refluxed with 10 times the volume of water for 60 min. Then, the extraction solution of single decoction was filtered and lyophilized (Beta 2–8 LDPlus, Christ, Germany) for use.

According to the dosage of CR and SR in the Huanglian Jiedu Decoction, 3.0 mg CR extraction and 2.0 mg SR extraction were accurately weighed in the ratio of 3:2, dissolved in the same amount of water, respectively. Then they were instantly mixed and prepared into 3.0 mg/mL stock solution (based on the concentration of CR) to obtain CR/SR mix. Due to the clinical practice of TCM, herb medicines’ decoction was usually boiled for 30 min. Based on the clinical practice and to clarify the difference of decocting, current physical mixture was decocted for 15 min, 30 min or 60 min to obtain CR/SR mix-15, CR/SR mix-30 or CR/SR mix-60, respectively.

### Preparation of phytochemicals self-assemblies

BBR and BA were weighed and dispersed with water, respectively. Then they were mixed instantaneously in the ratio of 1:1 to prepare the reserve solution of 5 μmol/mL, namely the physical combination solution of BBR-BA (BBR/BA mix). The above-mentioned physical mixture was decocted for 30 min or 60 min to obtain BBR/BA mix-30 or BBR/BA mix-60.

### Chemical composition analysis of the self-assembled parts from TCM decoctions

A high-performance liquid chromatography (Agilent Technologies, USA) method was used to compare the main chemical components of herbal decoctions. Equipping with an Ultimate LP-C18 column (4.6 mm × 250 mm, 5 μm), being maintained at 25 °C The flow rate of mobile phase was maintained at 1.0 mL/min with 0.2% (*v/v*) aqueous phosphoric acid solution (A) and acetonitrile (B). The gradient elution conditions were set as follows: 0–10 min, 5%-15% B; 10–18 min, 15%-23% B; 18–38 min, 23%-35% B; 38–45 min, 35%-40% B; 45–50 min, 40% − 90% B; 50–60 min, 90% B; 60–62 min, 90%-5% B; 62–68 min, 5% B. The injection volume was 10 μL.

### Characterization of the self-assembled parts

Field emission scanning electron microscopy (FESEM, ZEISS-SUPRA55, Germany) was used to measure the morphology of the self-assemblies, and dynamic light scattering (Zetasizer Nano ZS 90, Malvern Instrument, UK) was used to measure the size distribution and potential of each sample. The ^1^H-NMR spectra of the samples were recorded by Avance IIIHD 400 MHz spectrometer (Bruker, Billerica, MA, USA). Other spectral characteristics were determined by Ultraviolet–visible spectroscopy (HITACHI UH5300, Japan), Fourier transforms infrared spectroscopy (NicoletiS10, Thermo, USA) and X-ray powder diffractometer (Bruker, Karlsruhe, Germany), respectively. At the same time, the chiral characteristics of the samples were observed by Circular dichroism spectroscopy (Chirascan V100, Applied Photophysics, UK).

### Bacterial culture and antibacterial activity

The antibacterial experiments against *S.aureus* (ATCC 6538P) were carried out in vitro by microbroth dilution method and plate counting method. In short, the prepared sample solution was added into the 48-well plate by micro-broth dilution method and continuously diluted in the medium for 5 concentration gradients; that is, the concentrations of each sample of the herbal solutions in nutrient broth medium were finally 0.18, 0.09, 0.045, 0.0225, 0.01125 mg/mL. Meanwhile, the extract of CR was taken as positive control, and the nutrient broth was used as negative control. The concentrations of each sample of the phytochemicals self-assemblies in nutrient broth medium were finally 0.1, 0.05, 0.025, 0.0125, 0.00625 μmol/mL. BBR was used as positive control, and the nutrient broth was used as negative control. Then the bacteria suspension with the same concentration (2 × 10^6^ CFU/mL, 40 μL) was added to each well, thoroughly mixed, the plate was placed in a thermostatic incubator at 37 ℃ for 16 h. The absorbance of the bacteria was measured at 600 nm (OD 600) with a microplate analyzer to observe and calculate the MIC of the samples. The value of MIC was defined as the minimum concentration of the sample at which the medium is free of turbidity. In the end, standard plate counting method was used to further confirm the MIC of each sample. Briefly, the bacterial suspension was diluted with normal saline 1 × 10^5^ times, then inoculated on nutrient AGAR medium, and cultured in thermostatic incubator at 37 °C for 16 h. The growth of bacterial colonies was observed and counted. Each concentration was repeated three times.

### Quantitative evaluation of biofilm by crystal violet assay

The bacterial biofilm removal ability of the samples was evaluated by standard crystal violet method. And the detailed preparation of *S.aureus* biofilm as supplementary material in the Supporting Information. After the biofilm sample was obtained, it was rinsed with PBS once or twice to remove the residual planktonic bacteria in the 48-well plate. Then the biofilm was added 500 μL 100% methanol and incubated for 15 min to fix. The methanol was removed, and the wells were dried. Crystal violet solutions (500 μL 1% wt) were added to stain for more than 30 min. Then the crystal violet solutions were washed thoroughly with distilled water. After this, the remaining biofilms were dissolved by adding 500 μL solution of 30% acetic acid (*v/v*) to the well, and then the solution in the well was transferred to the new one, which was measured at 595 nm (OD 595) with a microplate analyzer to quantitatively evaluate the stained biofilm.

### Morphological observation of bacteria

FESEM was used to observe the morphological changes of the samples treated with bacteria. According to the above experimental method of bacterial culture, the 48-well plate was cultured in a constant temperature incubator at 37 °C for 8 h. Then the bacterial liquid of each sample was collected in different centrifuge tubes, centrifuged at 3000 r/min for 10 min, and the bacteria were collected. The glutaraldehyde (2.5%, *v/v*) was added to fix for 4 h after gently washing triple with PBS. And then eluted with ethanol solution with gradient concentration of 30%, 50%, 70%, 80%, 85%, 90%, 95% and 100%, respectively, and dehydration was conducted for 10 min each time. The bacteria were dispersed with an appropriate amount of distilled water after fully drying. Next, dropped onto a clean silicon plate to obtain the final bacterial sample. In the end, they were sputtered-gilded and observed with FESEM.

### Morphological observation of the biofilm

FESEM was used to further observe the removal effect of the samples on bacterial biofilm. That is, according to the above experimental method of bacterial biofilm culture, the biofilm was cultured on silicon wafer (5 mm × 5 mm) and placed in a constant temperature incubator at 37 °C for 24 h. Adding 0.4 μmol/mL of BBR/BA mix, BBR/BA mix-30 and BBR/BA mix-60, respectively. BBR was used as positive control, and blank culture medium was used as negative control. After culturing in thermostatic incubator at 37 °C for 24 h, the bacterial suspensions were absorbed and gently washed with PBS for 3 times. The glutaraldehyde with volume fraction of 2.5% was added and fixed for 4 h. Then it eluted with ethanol solution with gradient concentration of 30%, 50%, 70%, 80%, 85%, 90%, 95% and 100%, respectively. And dehydration was conducted for 10 min each time. Finally, the samples were sputter-coated by gold after fully drying and observed with FESEM.

### Evaluation of biofilm clearance by CLSM

The confocal laser scanning microscopy (CLSM) further confirmed the difference of biofilm scavenging ability between different samples. The biofilm samples were stained with live/dead BacLight bacterial viability kit (Thermo Fisher, Waltham, MA, USA) for 20 min at room temperature in darkness, and the biofilms were observed under a CLSM (Leica Microsystems, Heidelbbrg, Germany). The data were processed to obtain 3D images.

### Statistical analysis

All experiments were repeated at least three times, and the data were expressed as mean ± SD. Statistical analysis was performed using SPSS software (version 20.0) for analysis of variance. The independent sample t test was used to evaluate the statistical difference and significance of two independent groups. *P < 0.05, **P < 0.01 and ***P < 0.001 were used to indicate statistically significant, very significant and highly significant, respectively.

## Results and discussion

### Self-assembly formation and morphological studies of the herb pair

**Fig. 2:**
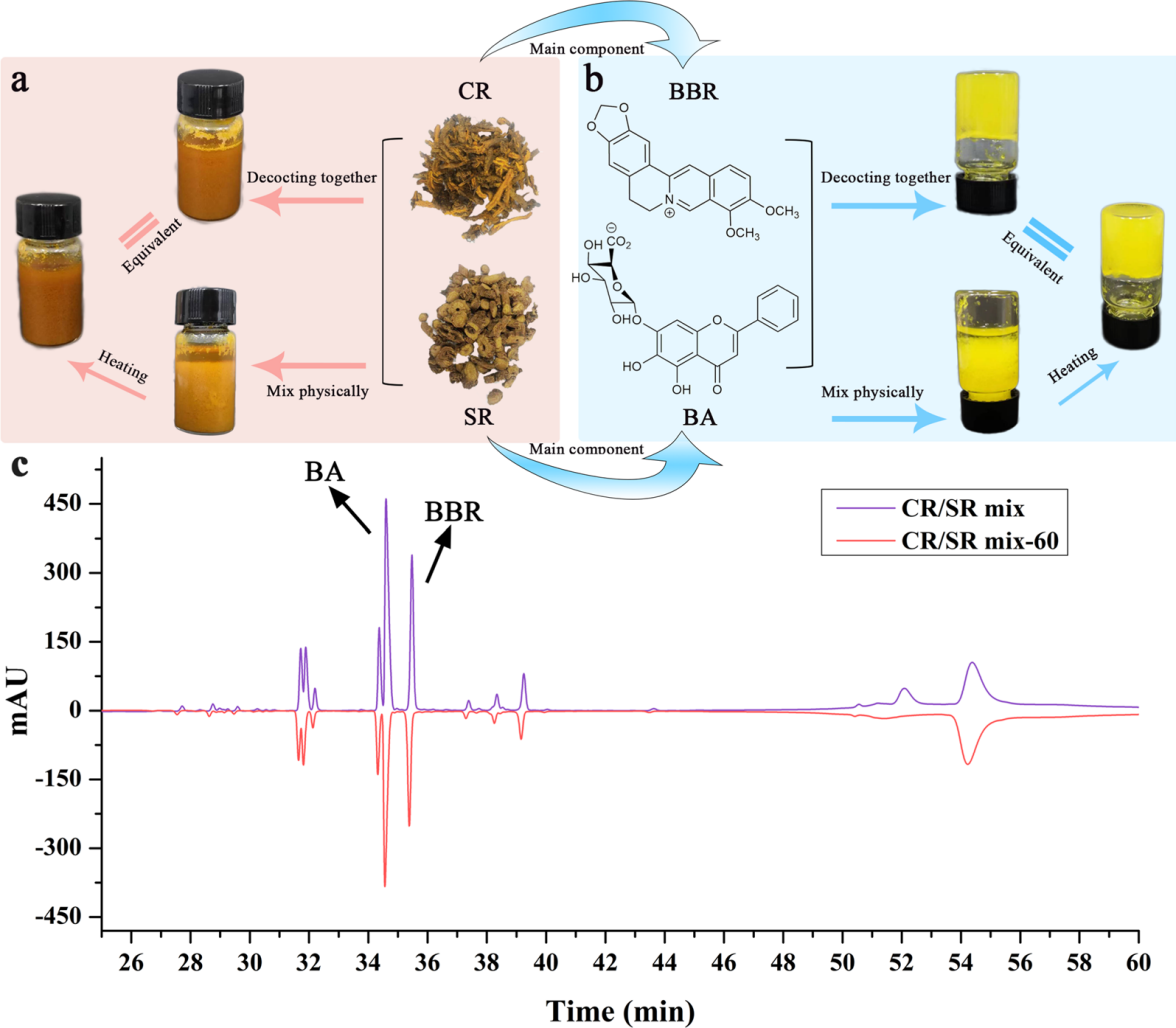
Macroscopical phenomena of co-decoctions and physical mixture; **a** The macro-state transitions of herbal decoctions was triggered by decocting; **b** The precipitation produced by physical mixture of BBR and BA was transformed into hydrogel by decocting; **c** Comparison of main components of CR/SR mix and CR/SR mix-60 (CR/SR mix-60 was prepared by decocted CR/SR mix for 60 min)

Combination of CR and SR was widely used in clinic. One way to prepare the herb pair was that they could be decocted singly just as herbal formula granules, then mixed before administration; the other way was that the herb pair were decocted together. Both the prepared methods were used in clinic. However, in the study of CR and SR, we found an interesting phenomenon that was the prepared methods had an enormous influence on the macro-state and micro-morphology of the decoction. As shown in Fig. 2a, obvious precipitation could be seen at the bottom of the single decoction physical mixture, while the co-decocted and re-heated physical mixture presented uniform suspension status. We found the micro morphology of these samples would change from a disorderly arrangement to a more uniform and orderly state during the decocting process. Obviously, compared with the physical mixture, the decoctions showed no obvious agglomeration phenomenon, and they were more uniform and better stability. Accordingly, the conversion of the macro-state and micro-morphology of the mixture and co-decoction was also occurred between BBR and BA, which were the main components of CR and SR. As shown in Fig. 2b, the results of the inverted test tube method showed that the physical mixture of BBR and BA formed obvious flocculent precipitation, while the reheated sample gradually formed a stable hydrogel. Furthermore, the main chemical constituents in the CR/SR co-decoction and the mixture were characterized using the high-performance liquid chromatography (Fig. 2c). It could be seen that both the co-decocted and physical mixture were roughly the same through comparison the detailed peaks, and the main components were BA and BBR. The result suggested that the difference in morphology was not caused by the production of new substances in the decocting process.

Our previous study found that BA and BBR could form NPs by electrostatic attraction, π-π stacking and hydrophobicity [24]. In this work, we discovered that the morphonology of BA-BBR self-assembly could be influenced by thermodynamics, which was accordance with the trend of uniform distribution of CR-SR. In other words, physical mixture samples could form a uniform system after decocting.

**Fig. 3:**
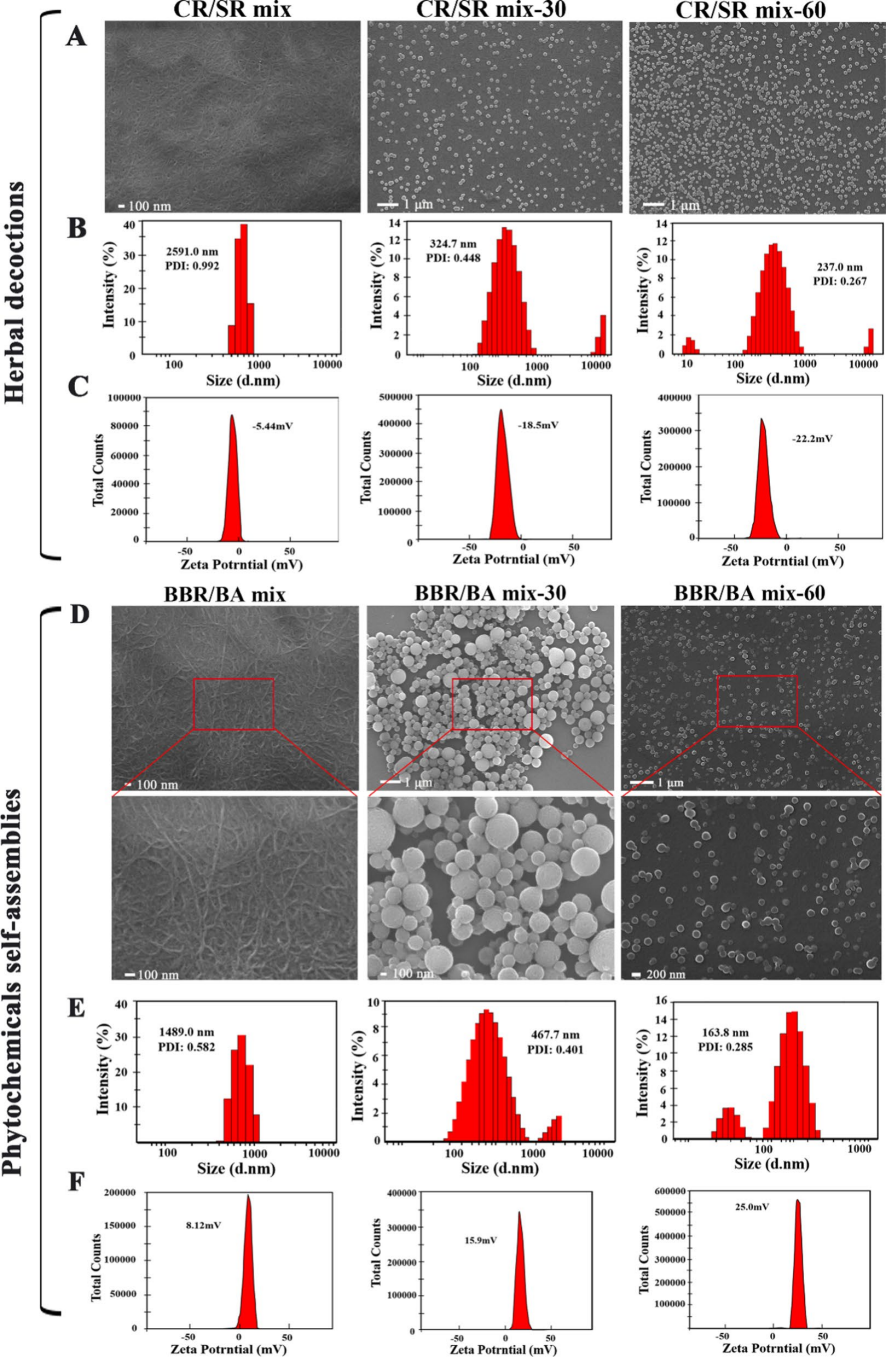
Micromorphology of self-assemblies; **a** FESEM images; **b** the particle size distribution and **c** the zeta potentials of the herbal decoctions; **d** FESEM images. **e** the particle size distribution and **f** the zeta potentials of the phytochemicals self-assemblies

To further study the thermodynamic process how to influence the CR and SR morphologies, the CR/SR mix was physically mixed the herb pair’s single decoction; then the mixture was reheated for reflux 30 min and 60 min to gain BBR/BA mix-30 and BBR/BA mix-60, respectively. The morphology of CR/SR mix was irregular NFs with a width of 100–200 nm and a length about 2–3 μm. It was worth noting that the BBR/BA mix-30 and BBR/BA mix-60’s morphologies of the assemblies were uniform and regular, and the diameters were gradually concentrated in approximately 200–350 nm to 100–250 nm with the extension of decocting time (Fig. 3a). In addition, the micro-morphology of CR/SR mix-15 also reflected this trend (Additional file 1: Fig. S1a). To explore the size distribution of the self-assembled parts, a dynamic light scattering (DLS) experiment was conducted. The results (Fig. 3b) suggested that the average diameter of CR/SR mix was around 2591.0 nm, the CR/SR mix-30 was around 324.7 nm, and the CR/SR mix-60 was around 237.0 nm, which were consistent with the results characteristics of FESEM. In the meantime, Zeta potential is commonly used to investigate the stability of the whole system, which could express the mutual attraction between particles very well. With the absolute value of Zeta potential increasing, the greater repulsive force between particles and the more stable of the system is created[31–34]. As shown in Fig. 3c, the absolute potential of CR/SR mix was the smallest (-5.44 mV). In contrast, the absolute values of Zeta potential of CR/SR mix-30 (-18.5 mV) and CR/SR mix-60 (-22.2 mV) increased significantly. This indicated that with the absolute value of Zeta potential of the assembly increased, and the decoction system became more stable.

We also monitored the micro morphology of the phytochemicals self-assemblies. The morphologies of monomer BBR and BA were obviously different from that of assemblies (Additional file 1: Fig. S2), and they did not show the properties of self-assembly. Intriguingly, various sizes of self-assemblies were formed under different decocting time, which was consistent with the trend of herbal decoctions. As shown in Fig. 3d and Fig. 3e, the field of view of the BBR/BA mix was filled with NFs with a width of more than 100 nm and a length of several microns. The morphology distribution of BBR/BA mix-60 was uniform, most of them were regular NPs with a particle size about 150–250 nm. Meanwhile, BBR/BA mix-15 (Additional file 1: Fig. S1b) and BBR/BA mix-30 seemed to be in the transition stages between these two forms, in which there were slightly wrinkled NPs with a diameter of 200–500 nm. Besides, the results of DLS were also in accordance closely with the information obtained from the FESEM images. The average diameter of the NPs changed from a few microns to about 100 nm, and the particle size distribution became more concentrated under the influence of thermodynamic parameters (thermal energy). Similarly, the absolute value of Zeta potential of BBR/BA mix was about 8.12 mV, while BBR/BA mix-30 (15.9 mV) and BBR/BA mix-60 (25.0 mV) increased significantly with the extension of decocting time (Fig. 3f). These phenomena further revealed the effect of decocting on the particle size, morphology and stability of self-assemblies. The above evidence preliminarily confirmed that thermodynamic process could affect the arrangement of herb pair self-assemblies.

### Mechanism of self-assemblies’ micromorphological change

Based on the consistency of the self-assemblies’ characters in herb pair CR-SR and its main component BBR-BA, we further discussed the mechanism of micromorphological change of BBR-BA self-assembly. Ultraviolet–visible (UV–vis) absorption spectrum, flourier transform infrared (FT-IR) spectrum, nuclear magnetic resonance hydrogen spectrum (^1^H-NMR), the X-ray powder diffraction pattern (XRD) and circular dichroism spectrum (CD) were used to study the spectral properties of the self-assembly. And the changes of the arrangement of BBR-BA molecular complex during the decocting process were preliminarily analyzed. Firstly, the results of UV–vis (Additional file 1: Fig. S3a) and FT-IR (Additional file 1: Fig. S3b) clearly showed that the characteristic absorption peaks of BBR/BA mix were the same as those of BBR/BA mix-30 and BBR/BA mix-60. Both of them revealed that the self-assembled unit of BBR/BA complex was stale and similar in different prepared protocols.

**Fig. 4:**
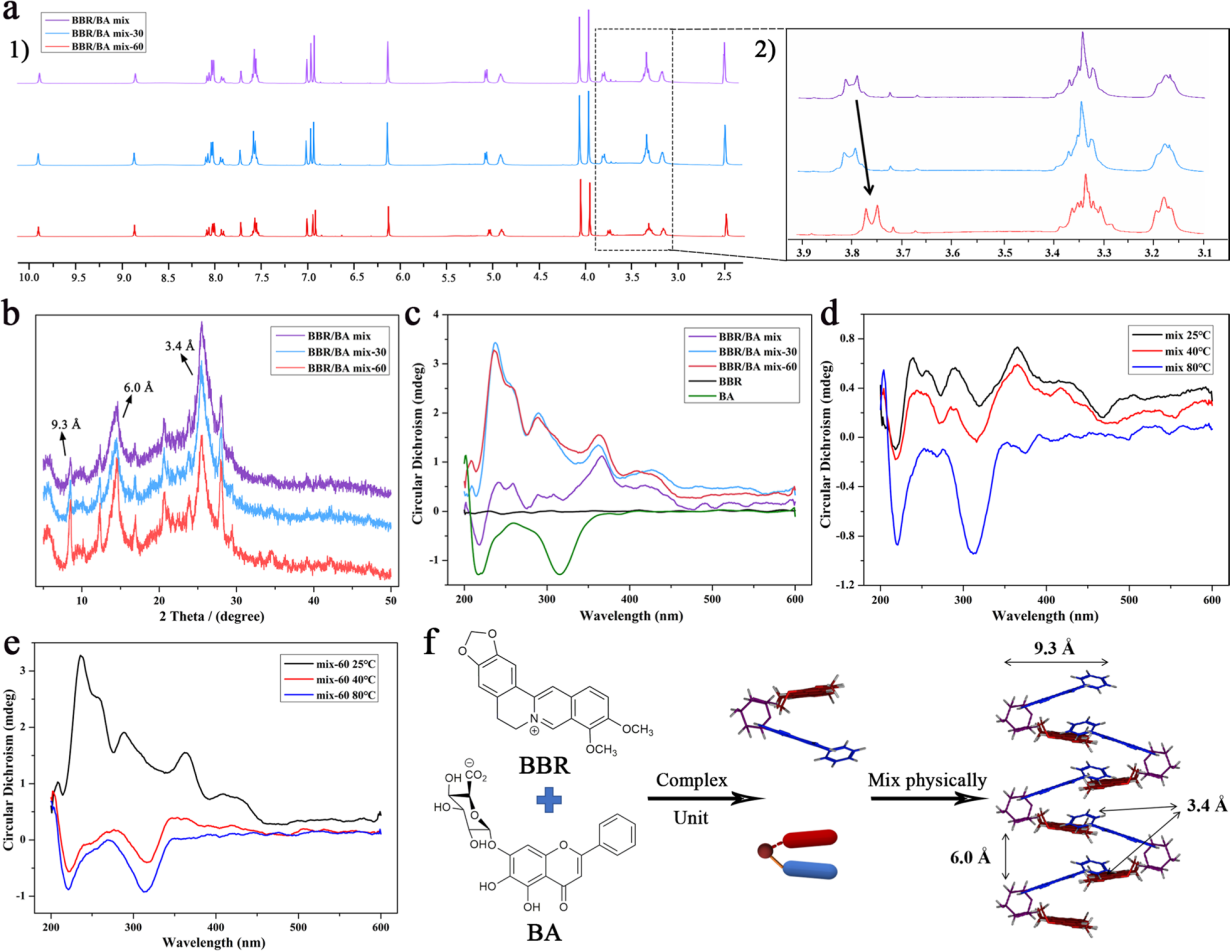
Characteristics of phytochemical self-assemblies; **a** 400 MHz ^1^H-NMR spectra. **b** XRD pattern of the phytochemicals self-assemblies; **c** CD spectra of BBR, BA and the three kinds of self-assemblies; **d**, **e** CD spectra of BBR/BA mix and BBR/BA mix-60 at different temperatures; (f) Spatial conformations of units of BBR/BA mix

XRD was usually used to analyze the spatial structure of molecules; and it could also be used for the semi-quantitative analysis based on the relative strength of the same diffraction peaks. XRD was applied to test the phytochemical samples and analyzed the molecular stacking pattern [35, 36]. According to the results (Fig. 4b), it could be clearly seen that in the process of interaction BBR and BA, the extension of decocting time did not lead to the change of the peak position of the characteristic signal peaks of the self-assembly, which also demonstrated that thermodynamic process could not change the essential properties of self-assemblies. Among them, 3.4 Å was the typical π-π accumulation characteristic peak, 9.3 Å was corresponded to the molecular horizontal length of BBR-BA complex measured by MOE software, and 6.0 Å was speculated to be the distance between adjacent glucuronic acid groups [24].

Notably, the relative strength of these characteristic peaks at the same diffraction angle had changed significantly. It was speculated that although they were roughly similar in structure, there might be some differences in the content of functional groups, and the change of assembly was mainly related to π-π accumulation. And according to the ^1^H-NMR data (Fig. 4a), it was found that the peak shape and chemical shift of the glycosyl group of BBR/BA mix-60 were slightly different from the other two samples, indicating that the spatial position of hydrogen atoms on the glycosyl group was different, which might be related to the arrangement of self-assembled molecules.

Moreover, CD spectrum was used to further explore the influence of thermodynamic on the optical rotation characteristics of each sample[37]. As shown in Fig. 4c, at the same concentration, BBR/BA mix showed a rather messy weak overall spectra and had a negative absorption peak between 200 and 250 nm. By contrast, the other two samples had positive absorption at this wavelength, and the intensity of the characteristic peak was also significantly enhanced. The characteristic peaks of BBR/BA mix-60 occurred with the shifts compared to BBR/BA mix (from 241 to 236 nm, from 366 to 362 nm). This was probably due to molecular rearrangement to form a close-knit self-assembly system, resulting in a conformational change in three-dimensional space. Just like certain proteins, the content of α-helix and β-helix and the interactions between secondary structures could be judged according to the difference and changing characteristics of the adsorption in CD [38, 39]. In accordance, as shown in Fig. 4d, it was found that when the heating temperature reached 80℃, the peak shape of BBR/BA mix gradually tended to be consistent with the CD spectrum of BA monomer solution, indicating self-assemblies would exist in the form of dissociative state at high temperature; In other word, the thermodynamics could affect the existence state of the self-assemblies. For BBR/BA mix-60 (Fig. 4e), decocting also dissociated the aggregation, and in the process of programmed cooling (from 80 °C to 40 °C and 25 °C), BBR-BA molecular complex would gather slowly again, changing from the negative cotton effect of dissociative state to the positive cotton of aggregated assemblies. And the intensity of the signal peak was larger than that of BBR/BA mix because the complete complexation of BBR and BA was promoted by thermodynamic factors. Thereafter, we confirmed that the internal arrangement of molecules was changed under the influence of thermodynamic parameters.

**Fig. 5:**
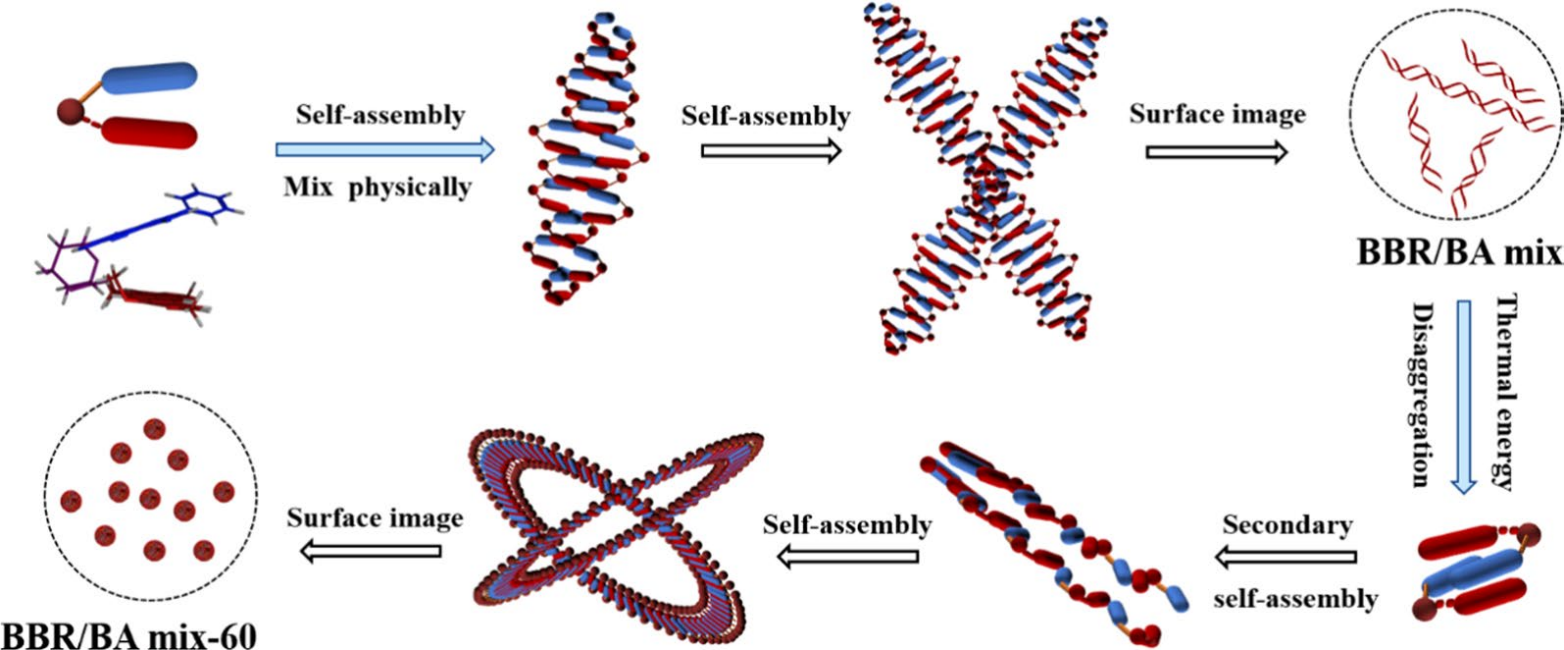
The schematic diagram of the self-assembly processes of BBR/BA mix (Precipitation state) and BBR/BA mix-60 (Hydrogel state)

Based on the above evidence and our previous study, it displayed that BBR and BA first formed amphiphilic structure of BBR-BA complex molecule driven by electrostatic gravity, and then took the amphiphilic complex molecule as the basic unit for further assembly [24]. The self-assembly mechanism of BBR/BA mix was as following: the hydrophobic parent nucleus of BA and BBR in BBR/BA mix were most likely to cross each other to form a stable system which was presented as flocculent precipitation; and they were arranged with hydrophilic groups facing outside and hydrophobic groups facing inside in water solution to agglomerate into flexible NFs, which was similar to the DNA double helix (Fig. 4f). After decocting BBR/BA mix, the aggregation state depolymerized and existed in a more active state driven by thermal energy. The weak ionization of glucuronic acid in BA was enhanced, which made the affinity between BBR and BA stronger. Therefore, BBR and BA interacted to obtain sufficient free energy association. As the temperature slowly decreased, the assembly units were rearranged to form the uniform NPs, which was a hydrogel and reached the thermodynamically stable low energy state [40, 41]. The assembly mechanism provided promising evidence for explaining the morphological differences between BBR/BA mix and BBR/BA mix-60 (Fig. 5). The result was similar to previous research that thermodynamic process led to morphological changes of self-assemblies. For example, it had been reported that diphenylalanine peptide could change the nanostructure under the induction of temperature, and finally transformed into a thermodynamically stable crystal structure [42]. And some experiments showed that heating could induce lipid-encapsulated bubbles to change from vesicles to smaller micelles, so that their ultrasound imaging capacity were better, and they could enter the small blood vessels in the tissue, which were suitable for drug delivery [43].

### Antibacterial assays

**Fig. 6:**
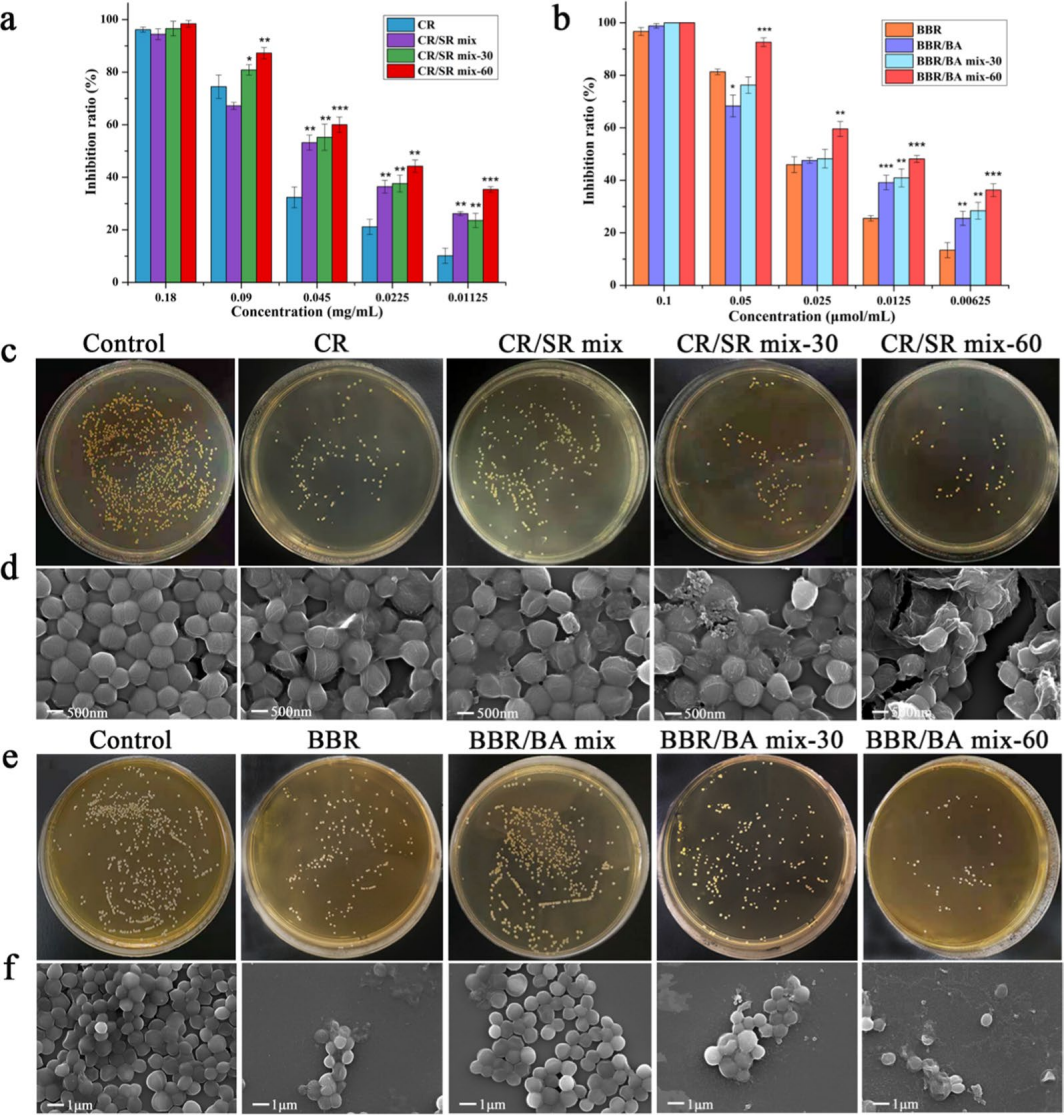
Bacteriostatic activity; **a**, **b** Inhibition rate of herbal decoctions and phytochemicals self-assemblies at different concentrations against *S.aureus*; **c**, **e** Bacterial colonies on culture medium treated with herbal decoctions and phytochemicals self-assemblies; **d**, **f** FESEM images of *S.aureus* incubated with herbal decoctions and phytochemicals self-assemblies

The influences of decocting time on the antimicrobial activity of different samples were preliminarily investigated by microbroth dilution method and standard plate counting method, respectively. As shown in Fig. 6a, b, the inhibitory effects of herbal decoctions and BA/BBR phytochemical self-assemblies on bacterial proliferation were in a dose-dependent manner. Interestingly, the antibacterial activity of the samples was also improved with the extension of decocting time. That was to say, the antibacterial potency of the samples enhanced with the changes of their morphologies from NFs to NPs. The data showed that at the concentration of 0.09 mg/mL, the bacteriostatic rate of CR/SR mix was only 65.96 ± 1.54%, while CR/SR mix-60 was 87.27 ± 2.14%. CR/SR mix-60’s activity was enhanced 1.32 times than that of CR/SR mix. Meanwhile, the results of plate counting (Fig. 6c) also proved that CR/SR mix-60 had the best inhibition effect on bacterial growth, serially followed by CR/SR mix-30 and CR/SR mix. At low concentration (< 0.05 mg/mL), the antimicrobial activities of all herbal decoctions were much better than that of the same concentration of CR and SR (Additional file 1: Fig. S4a).

The same results were also confirmed in the phytochemical self-assemblies simulated by BA and BBR monomer components. Microbroth dilution method demonstrated that the antibacterial activity trend of phytochemicals self-assemblies was consistent with that of the herbal solutions. For example, at 0.05 μmol/mL concentration, BBR/BA mix-60 had the strongest antibacterial effect (92.63 ± 1.64%) than BBR/BA mix-30 (76.26 ± 3.15%) and BBR (81.32 ± 1.10%), respectively. In contrast, BBR/BA mix (68.32 ± 4.17%) had the weakest effect on *S.aureus*. The antimicrobial potency of BBR/BA groups at low concentration (< 0.05 μmol/mL) was also better than that of BBR and BA (Additional file 1: Fig. S4b). Likewise, the standard plate counting method was used to verify the activity of the samples. The antibacterial effects of the four samples at the concentration of 0.05 μmol/mL were shown in Fig. 6e. Among them, the BBR/BA mix-60’s antimicrobial activity was better than BBR/BA mix, which was in accordance with the CR/SR’s results and manifested the antibacterial activity of the physical mixture could be significantly improved after decocting.

To further evaluate the influence of bacteriostatic activity of herbal decoctions during thermodynamic process, FESEM was used to observe the morphological changes of *S.aureus* treated at same concentration. The bacteria in control group presented a perfect spherical shape with smooth surface and no damage (Fig. 6d). As for the herbal decoctions, the sizes of the bacteria treated by CR, CR/SR mix, CR/SR mix-30 and CR/SR mix-60 were obviously different, and the cell walls of the bacteria were all depressed and shrunk. Among them, the bacterial surface began to shrink and sag, and part of the bacterial morphology was destroyed after the treatment with CR/SR mix-30; The intervention of CR/SR mix-60 would lead to the serious destruction of cell membranes integrity, great changes in bacterial morphology and even widespread rupture. In contrast, the morphology changed slightly after the treatment of CR and maintained regular ellipsoid; the cell membrane damage degree was small and only local rupture with the treatment of CR/SR mix.

Meanwhile, the antibacterial effects of the phytochemicals self-assemblies could be clearly observed from the Fig. 6F. BBR/BA mix-60 treatment could cause obviously changes in bacterial morphology, such as surface damage and incomplete cell membrane structure. Of note, compared with BBR/BA mix, BBR/BA mix-30 and BBR/BA mix-60 had more ruptured bacteria with irregular shape and rough surface appeared. As a result, thermal energy could improve the activity of the samples by influencing the morphology of self-assemblies.

### Eradicating *S.aureus* mature biofilms

**Fig. 7:**
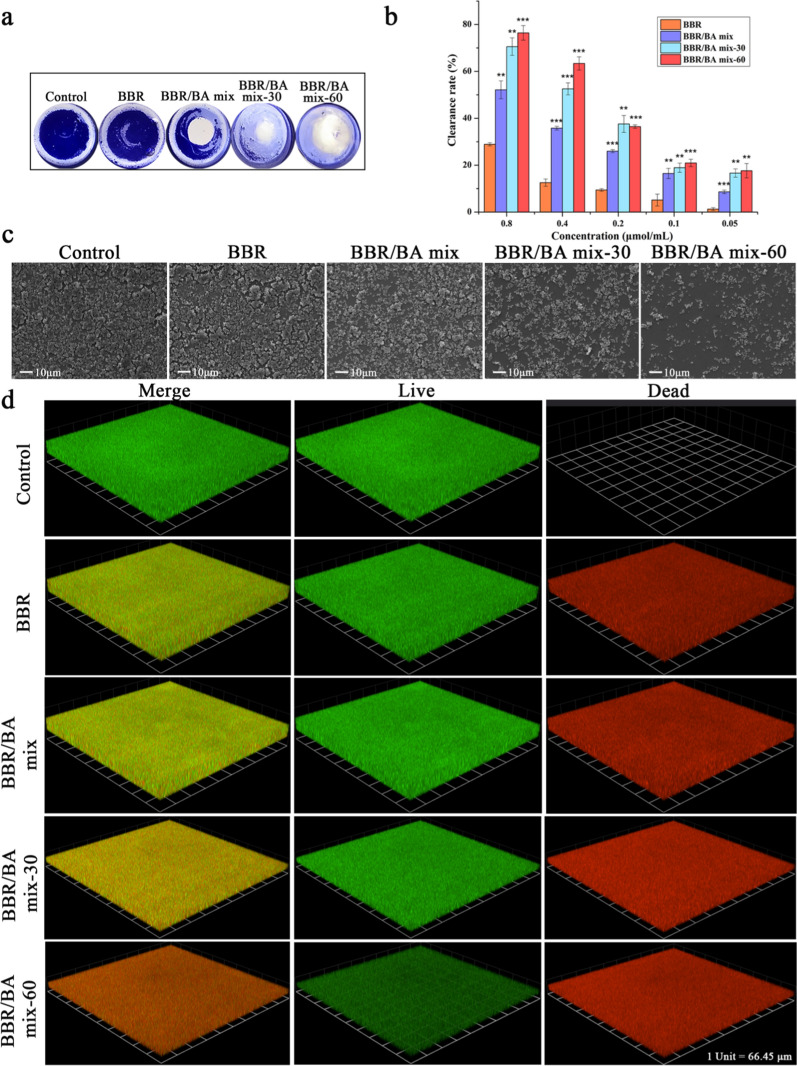
**a** Crystal violet-stained biofilm; **b** Biofilm clearance rates of BBR/BA mix, BBR/BA mix-30 and BBR/BA mix-60; (**c**) FESEM images of biofilms treated by BBR/BA mix, BBR/BA mix-30 and BBR/BA mix-60; **d** 3D images of normal *S.aureus* biofilm and *S.aureus* biofilm treated with phytochemicals self-assemblies

The bacterial biofilm is a complex community formed by extracellular matrix composed of proteins, polysaccharides and extracellular DNA and inner bacterial plankton, which is one of the main drug resistance mechanisms of *S.aureus*. BBR/BA self-assembly can resist the attack of phagocytic cells or effectively block the infiltration of antibiotics, leading to bacterial drug resistance [44, 45]. We further investigated whether thermodynamic process affected the antibacterial activity of phytochemical self-assemblies through the scavenging experiment of bacterial biofilm. The images of crystal violet staining (Fig. 7a) showed that the treatment effect of BBR/BA mix was weak, while the amount of biofilm residue was lessened after co-decocting. As presented in Fig. 7b, at 0.8 μmol/mL concentration, the eradicating rates of BBR/BA mix-60 and BBR/BA mix-30 were 76.43 ± 3.11% and 70.56 ± 3.74%, respectively. On the contrary, the eradicating rate of BBR/BA mix was only 52.16 ± 3.83%; BBR had the least eradicating activity, which had little effect on biofilm, only 28.92 ± 0.68%. In addition, it was obvious that the activity of the decocted samples was better than that of the physical mixture, which was consistent with the above bacteriostatic experiment results. Moreover, the biofilm treated by BBR/BA mix-60 was thinnest and sparsest, and the cell rupture was serious.

FESEM was also employed to validate the treatment of different samples on *S.aureus* biofilms (Fig. 7c). The scavenging effect of BBR on biofilm was weak; and the biofilm treated with BBR/BA mix became thinner slightly, but the morphology of bacteria was intact. On the contrary, the distribution of bacteria in BBR/BA mix-30 and BBR/BA mix-60 was significantly less than that in BBR/BA mix and BBR; among which, BBR/BA mix-60 was the least, indicating that the activity of the samples could be optimized by decocting. The changes of biofilm could be further confirmed through fluorescence staining assays. Green fluorescence represents living bacteria, while red fluorescence represents dead bacteria. The redder of the bacteria indicated the more dead bacteria. In Fig. 7d, the untreated group only fluoresced brightly in green, and after 24 h incubation with BBR or BBR/BA mix, the number of dead bacteria increased slightly. In contrast, the biofilm treated by BBR/BA mix-60 was thinnest and sparsest, which displayed the brightly red fluorescence and weakly green fluorescence. This result was the same as the above crystal violet staining result and FESEM images, which further confirmed that with the appropriate prolongation of decocting time, self-assemblies could cause more significant damage to bacterial cell walls and membranes.

### Sequencing of the *S.aureus* transcriptome

**Fig. 8:**
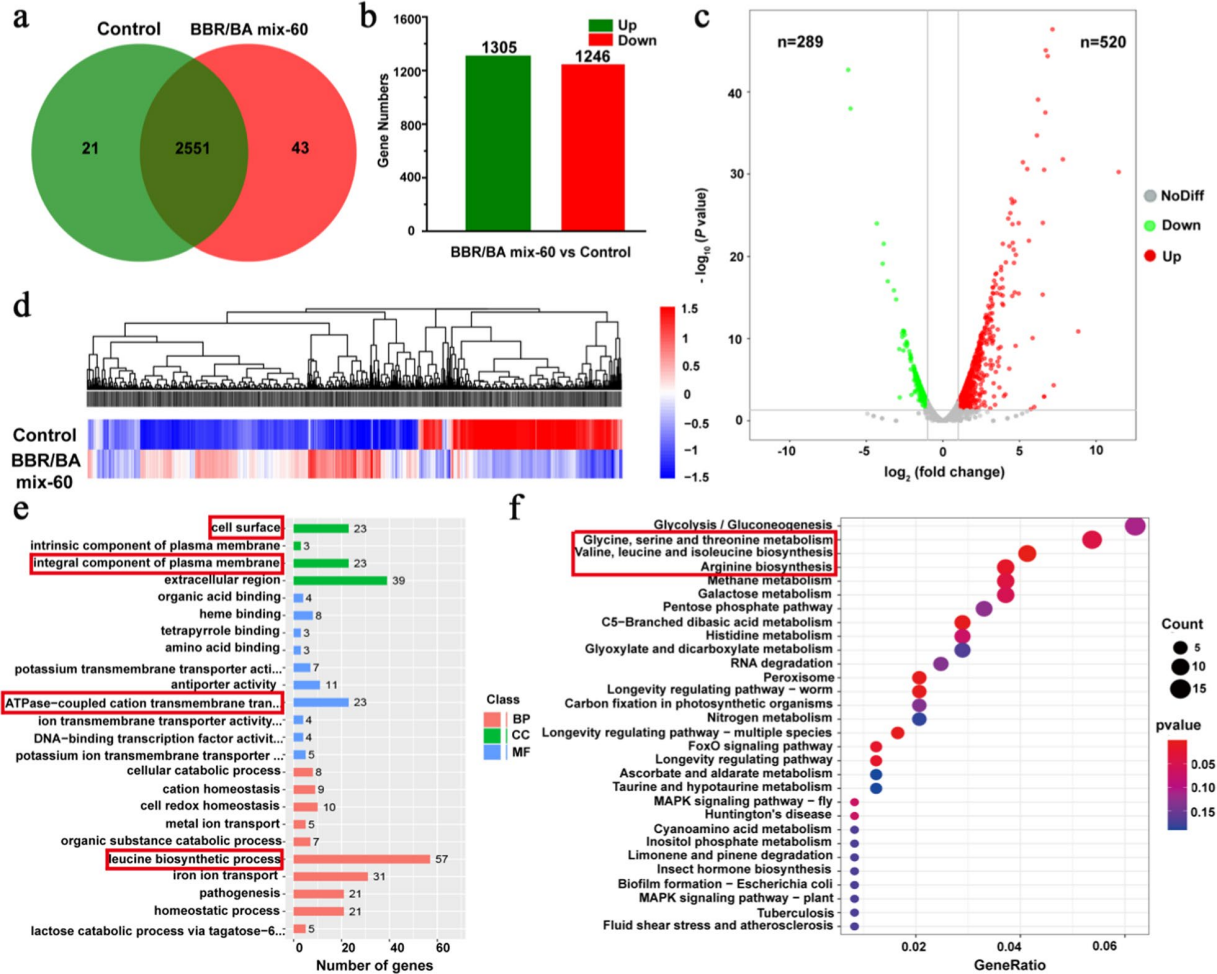
RNA-seq analysis. **a** Venn diagram of the Genes in Control and BBR/BA mix-60. **b** Up-regulation and down-regulation of genes in Control vs BBR/BA mix-60. **c** Volcano plot showing differentially expressed genes (DEGs) in Control vs BBR/BA mix-60. **d** Hierarchical clustering analysis of DEGs in Control and BBR/BA mix-60 groups. (e) GO enrichment analysis of DEGs in BBR/BA mix-60. **f** KEGG pathway enrichment analysis of DEGs in BBR/BA mix-60

Through morphological studies and activity tests, we had known that the morphology of self-assembly changed from NFs to NPs driven by thermal energy; BBR/BA mix-60 with homogeneous NPs had the strongest antibacterial activity and biofilm scavenging effect. To gain deeply insight into the mechanisms of BBR/BA mix-60 against *S.aureus* at the genetic level, Illumina RNA-seq was further used to compare the transcriptome profile of bacteria treated with BBR/BA mix-60 and control group. The data showed that 2,572 genes, 2,594 genes were identified in the control and BBR/BA mix-60 groups, respectively (Fig. 8a). Compared with control group, 2,551 genes were shared. Moreover, 1,305 genes were upregulated, and 1,246 genes were downregulated (Fig. 8b). Then we focused on differentially expressed genes (DEGs) between the control and BBR/BA mix-60. As shown in Fig. 8c, a total of 809 DEGs were found, including 520 up-regulated genes and 289 down-regulated genes (*p* < 0.05, cut-offs of fold change > 2). At the same time, hierarchical clustering analysis showed that the majority of DEGs had reverse expression patterns in BBR/BA mix-60 group vs control group (Fig. 8d). The Gene Ontology (GO) enrichment analysis showed that DEGs were mainly enriched in cell surface, integral component of plasma membrane, ATPase-coupled cation transmembrane transporter activity and leucine biosynthetic process (Fig. 8e). The Kyoto Encyclopedia of Genes and Genomes (KEGG) pathway enrichment analysis showed that DEGs were mainly enriched in glycine, serine and threonine metabolism pathway, valine, leucine and isoleucine biosynthesis pathway and arginine biosynthesis pathway (Fig. 8f). These results showed that BBR/BA mix-60 mainly affected cell surface, amino acid biosynthesis and metabolism to effectively inhibit bacteria. This was very consistent with the results of FESEM that BBR/BA mix-60 seriously destroyed the morphology of bacteria. According to RNA-seq sequencing analysis, we took differentially expressed genes *nagB*, *mntA* and *mntC* as examples to explore the regulatory mechanism of BBR/BA MIX-60 on *Staphylococcus aureus* (Additional file 1: Fig S5 and Additional file 1: Table S1). Briefly, both BA and BBR could bind amino acid fragments on *nagB*, *mntA* and *mntC* with high affinity through weak bonding. It also affected the amino sugar decomposition and peptidoglycan biosynthesis of bacteria. In addition, the manganese ion transporter was downregulated to reduce the immune escape and attributed to its well antibacterial activity.

## Conclusion

In this study, we found that the physical mixture of both herbal medicine and phytochemical components would instantly precipitate to form large size NFs. Furthermore, the NFs could transform to NPs by the co-decocting; and NPs was more regular and stable, which was due to the temperature could affecting the arrangement of the assembly. Driven by the thermal energy, BBR and BA interacted with each other to obtain enough free energy association; Then the amphiphilic complex molecules were converted and assembled to form NPs under the guidance of weak bond forces; Thereafter, self-assemblies reached the thermodynamically stable low-energy state. The bacteriostatic test demonstrated that the antibacterial activity and clearing biofilm effect could be enhanced with the change of morphology from NFs to NPs. And this might be attributed to that NPs could obviously affected cell surface, then influenced amino acid biosynthesis and metabolism from RNA-seq test. The phenomenon was similar to the relationship between crystalline drugs and amorphous drugs; both of them have the same chemical structure, the same administration dose, but due to different molecular arrangements, resulting in different biological effects.

In summary, we found that the effective ingredients’ composition in herb medicine CR/SR decoction were equal to their simple mixtures, but they had different antibacterial activities. Clinical practice had displayed that the co-decocting of herbals could not only enhance efficacy, but also promote the water dissolution of active ingredients, eliminate or reduce toxicity [46–48]. This work exhibited that co-decocting herbal formula granules before taking might be beneficial to the treatment of disease, providing evidence for its scientific and effective application in clinical practice.

## Supplementary Information

**Additional file 1**: **Figure S1.** (a) FESEM images of BBR; (b) FESEM image of BA. **Figure S2**. (a) FESEM images of CR/SR mix-15; (b) FESEM images of BBR/BA mix-15. **Figure S3.** (a) The superimposed UV absorption spectroscopy of phytochemicals self-assemblies; (b) Fourier-transform infrared spectroscopy spectra of phytochemicals self-assemblie. **Figure S4.** Bacteriostatic activity; (a) Inhibition rate of SR and BA at different concentrations against S.aureus; (b) Bacterial colonies on culture medium treated with SR and BA. **Figure S5.** Molecular docking study. A. Baicalin with nagB test; B. Baicalin with mntC test; C. Baicalin with mntA test; D. Berberine with nagB test; E. Berberine with mntC test; F. Berberine with mntA tes. **Table S1**. Binding energy in molecular bocking.

## Data Availability

All data generated and analyzed during this research are included in this published article.
