# Supplementary material for: Thermodynamics driving phytochemical self-assembly morphological change and efficacy enhancement originated from single and co-decoction of traditional chinese medicine
Source: J Nanobiotechnology. 2022 Dec 12;20:527. doi: 10.1186/s12951-022-01734-w (PMC9743513; doi:10.1186/s12951-022-01734-w)
Supplement: Supplementary file 1 — Additional file 1: Figure S1. (a) FESEM images of BBR; (b) FESEM image of BA. Figure S2. (a) FESEM images of CR/SR mix-15; (b) FESEM images of BBR/BA mix-15. Figure S3. (a) The superimposed UV absorption spectroscopy of phytochemicals self-assemblies; (b) Fourier-transform infrared spectroscopy spectra of phytochemicals self-assemblie. Figure S4. Bacteriostatic activity; (a) Inhibition rate of SR and BA at different concentrations against S.aureus; (b) Bacterial colonies on culture medium treated with SR and BA. Figure S5. Molecular docking study. A. Baicalin with nagB test; B. Baicalin with mntC test; C. Baicalin with mntA test; D. Berberine with nagB test; E. Berberine with mntC test; F. Berberine with mntA tes. Table S1. Binding energy in molecular bocking. [file 12951_2022_1734_MOESM1_ESM.doc]

**Supporting Information for**

**Original Article**

**Thermodynamics Driving Phytochemical Self-assembly Morphological Change and Efficacy Enhancement Originated from Single and Co-decoction of Traditional Chinese Medicine**

Xuemei Huang^†^, Xiaojing Liu^†^, Xiaoyu Lin, Zhihua Yuan, Yaozhi Zhang, Zhijia Wang, Wenmin Pi, Haoqiang Zhao, Haimin Lei, Penglong Wang*

*School of Chinese Pharmacy, Beijing University of Chinese Medicine, Beijing 102488, China*

*Corresponding authors. Tel./fax: +86 10 84738641.

E-mail addresses: wpl581@126.com (Penglong Wang)

†Xuemei Huang and Xiaojing Liu contributed equally to this work

**1.FESEM Analysis of Self-Assemblies.**

The supramolecular system extracted from the herbal decoctions and phytochemicals self-assemblies were dispersed evenly with a small amount of deionized water. Then absorbed 3 μL, dripped gently onto the silicon wafer and dried naturally at room temperature. The gold film was subsequently coated with a LEICA-EM-ACE600 sputter coater instrument (Leica, Germany). Finally, the morphology of the self-assemblies was imaged on a FESEM (ZEISS-SUPRA55, Germany) operated at 8 kV.

**2.Particle Size Measurement.**

After diluting the self-assemblies with a small amount of deionized water, 1 mL was absorbed into a colorimetric dish. And its particle size and potential were measured by Malvern particle sizer (DLS, Zetasizer Nano ZS 90, Malvern Instrument, UK) at 25 °C. After three parallel measurements, both the average particle size and potential were recorded, respectively.

**3.Ultraviolet-Visible Spectroscopy (UV).**

The prepared BBR-BA self-assemblies with different decocting time were diluted with a small amount of deionized water and packed into a washed colorimetric dish. At the same time, deionized water was used as the reference solution. The UV spectra of samples were determined using a UV-visible Spectrophotometer (HITACHI UH5300, Japan) with the scanning range from 200 to 600 nm.

**4.Fourier Transform Infrared Spectroscopy (FT-IR).**

The BBR-BA self-assemblies with different decocting time were grinded evenly with a clean and dry mortar, and the quantitative freeze-dried powder was obtained. The single-channel scanning was carried out with the air as the background. And then the samples were placed in the sampling area for measurement by the FT-IR spectrometer (ALPHA II, Bruker, US) in the range from 4000 to 400 cm^-1^.

**5.Proton Nuclear Magnetic Resonance** **Spectroscopy of** ^1^**H-NMR.**

^1^H-NMR spectra were recorded on an Avance IIIHD 400 MHz spectrometer (Bruker, America) with tetramethylsilane as an internal standard. Monomer components (10 mg) and lyophilized powder of self-assemblies (15 mg) were dissolved by 1 mL DMSO-*d_6_*. The 1H-NMR of self-assemblies were analyzed by referring the ^1^H-NMR of monomer compounds.

**6.X-ray powder diffractometer**

The lyophilized powders of different self-assemblies (15 mg) were measured by PXRD at 40 kV and 40 mA on Rigku Ultima IV diffractometer and irradiated by Cu-Kα at room temperature in the range of 5 °- 50 °.

**7.Circular Dichroism Spectroscopy**

The BBR-BA self-assemblies with different decocting time were diluted with a small amount of deionized water for 5 times. The data were measured at the set temperature of 25 ℃, 40 ℃ and 80 ℃ by Circular dichroism spectroscopy (Chirascan V100, Applied Photophysics, UK) with the scanning range from 200 to 600 nm.

**8.Biofilm formation and destruction.**

The biofilm was cultured in 48-well plates according to the method established earlier in our laboratory. The bacterial suspension was removed to obtain the mature biofilm, and 0.8, 0.4, 0.2, 0.01 and 0.05 μmol/mL of BBR/BA mix, BBR/BA mix-30 and BBR/BA mix-60 were added, respectively. Meanwhile, BBR was used as positive control, and blank culture medium was used as negative control. After 24 h incubation in an incubator at 37℃, the final biofilm samples were obtained.

**9.Antibacterial Assays.**

The freeze-dried self-assemblies were dissolved with deionized water to a concentration of 3.0 mg/mL or 2.0 μmol/mL, then sample solution was added to the first well of a 48-well plate and diluted with nutrient broth to 0.18 mg/mL or 0.1 μmol/mL. Preparation methods of other concentrations were the same.

**10. RNA-Seq analysis.**

The *S.aureus* was exposed to BBR/BA mix-60 at 37°C. Total RNA was isolated from *S.aureus* using the mirVana miRNA Isolation Kit (Ambion) according to the manufacturer’s protocol. RNA integrity number (RIN) and quality were evaluated using the Agilent 2100 Bioanalyzer (Agilent Technologies, Santa Clara, CA, USA). It was generally believed that samples with RIN values higher than 7.0 meet the requirements of RNA-seq and could be directly used for subsequent database analysis. The cDNA libraries were constructed using the Illumina TruSeq RNA Sample Preparation kit following the manufacturer’s instructions. The quality and quantification of cDNA libraries were performed by using Agilent 2100 Bioanalyzer (Agilent Technologies, Singapore). The libraries were then sequenced using HiSeq Illumina 2500 sequencing platform. Functional pathway analyses of differentially expressed genes were performed using the Gene Ontology (GO) and Kyoto Encyclopedia of Genes and Genomes (KEGG) database.

**11.Molecular docking process**

The Mol2 structures of baicalin and berberine were downloaded from Lab of System Pharmacology (https://old.tcmsp-e.com/tcmsp.php). AutoDock Tools software was used to determine the rotation center and rotation bond of the ligands (baicalin and berberine). The crystal structure of the *mntA* (PDB code: 5HX7), *mntC* (PDB code: 4NNO), *nagB* (PDB code: 2RI0) were downloaded from the RCSB Protein Data Bank (www.rcsb.org). The three crystal structures were processed using PyMol Molecular Viewer (http://www.pymol.org/) and AutoDock Tools as follows: redundant protein conformations were deleted, water molecules were deleted, hydrogenated and associated charges were allocated. The docking range of the processed receptors and ligands was set by AutoDock Tools software, and the potential molecular binding mode was evaluated by AutoDock Vina software. Finally, PyMOL software and Discover Studio software were used to visually analyze the results with the highest docking score.

**Supporting figures and tables**

**
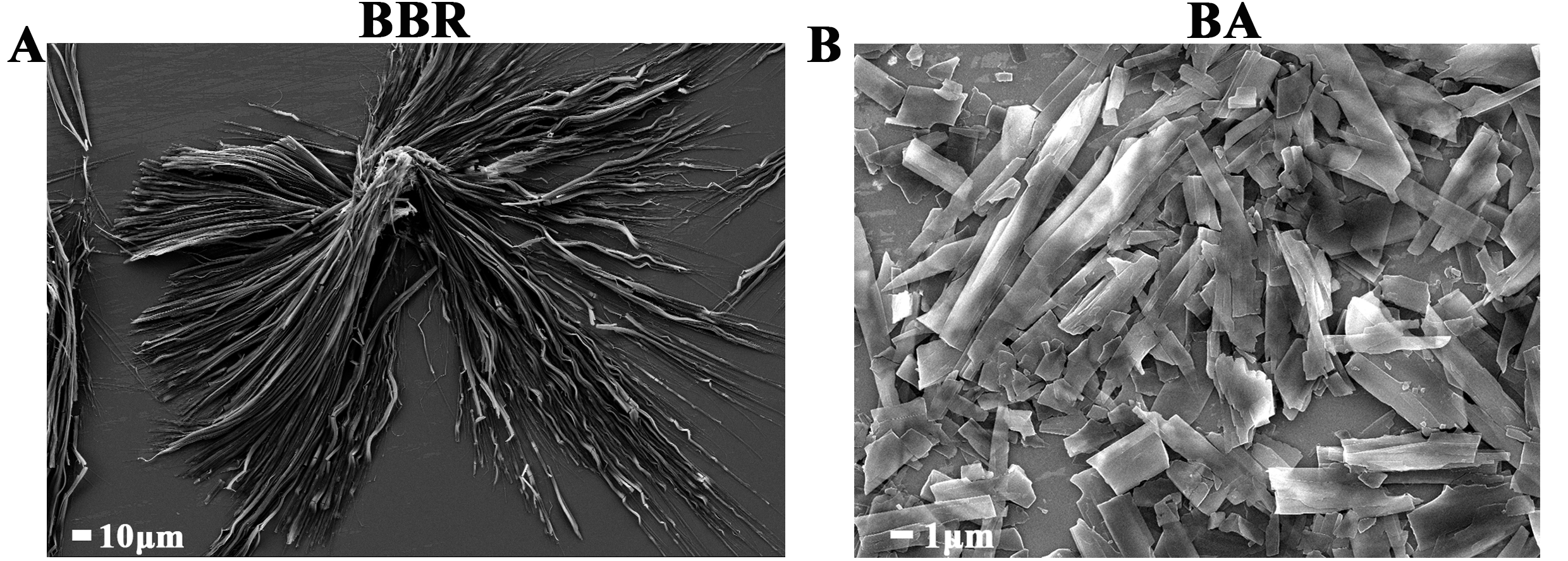
**

**Fig. S1** (a) FESEM images of BBR; (b) FESEM image of BA.


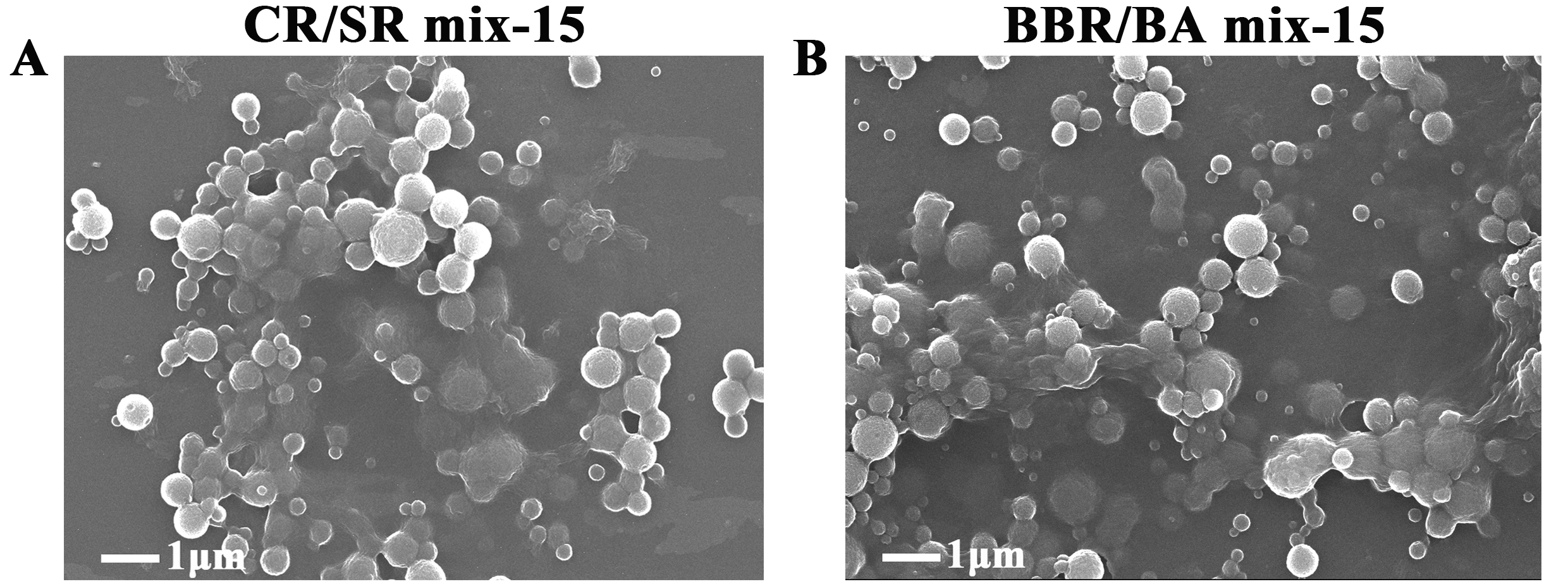


**Fig. S2** (a) FESEM images of CR/SR mix-15; (b) FESEM images of BBR/BA mix-15.


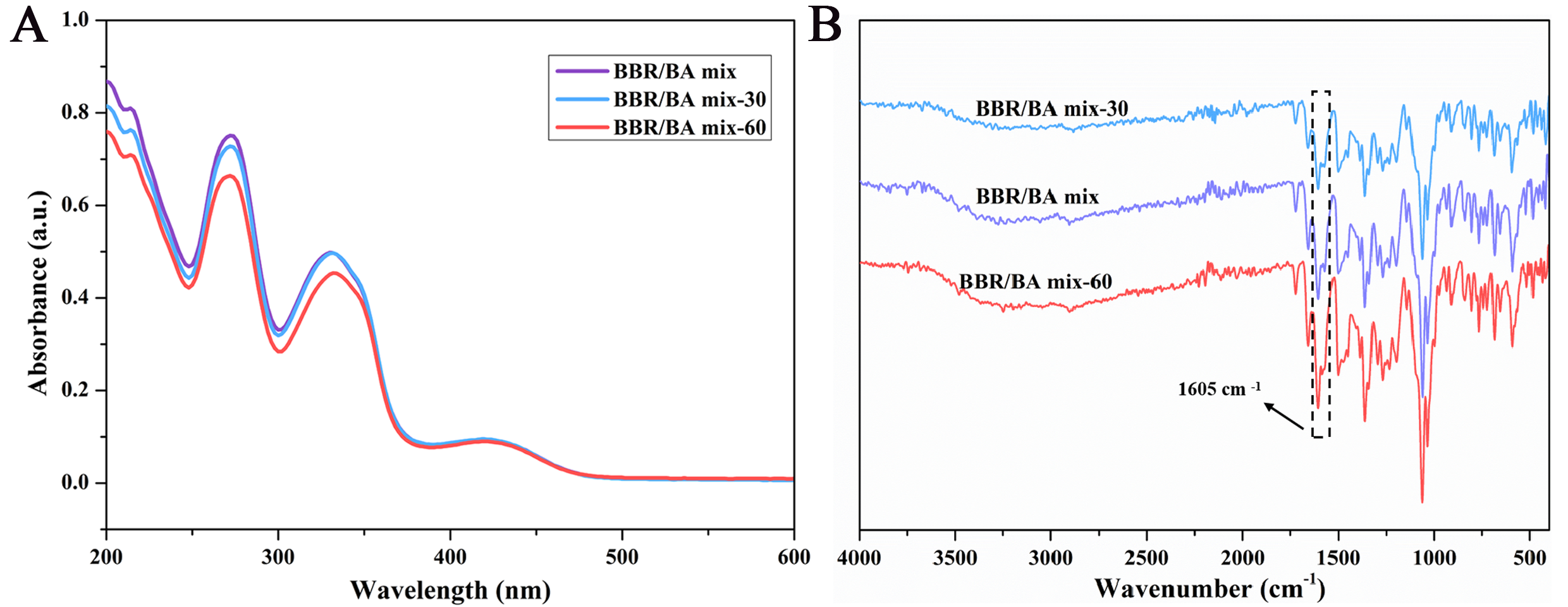


**Fig. S3** (a) The superimposed UV absorption spectroscopy of phytochemicals self-assemblies; (b) Fourier-transform infrared spectroscopy spectra of phytochemicals self-assemblies.


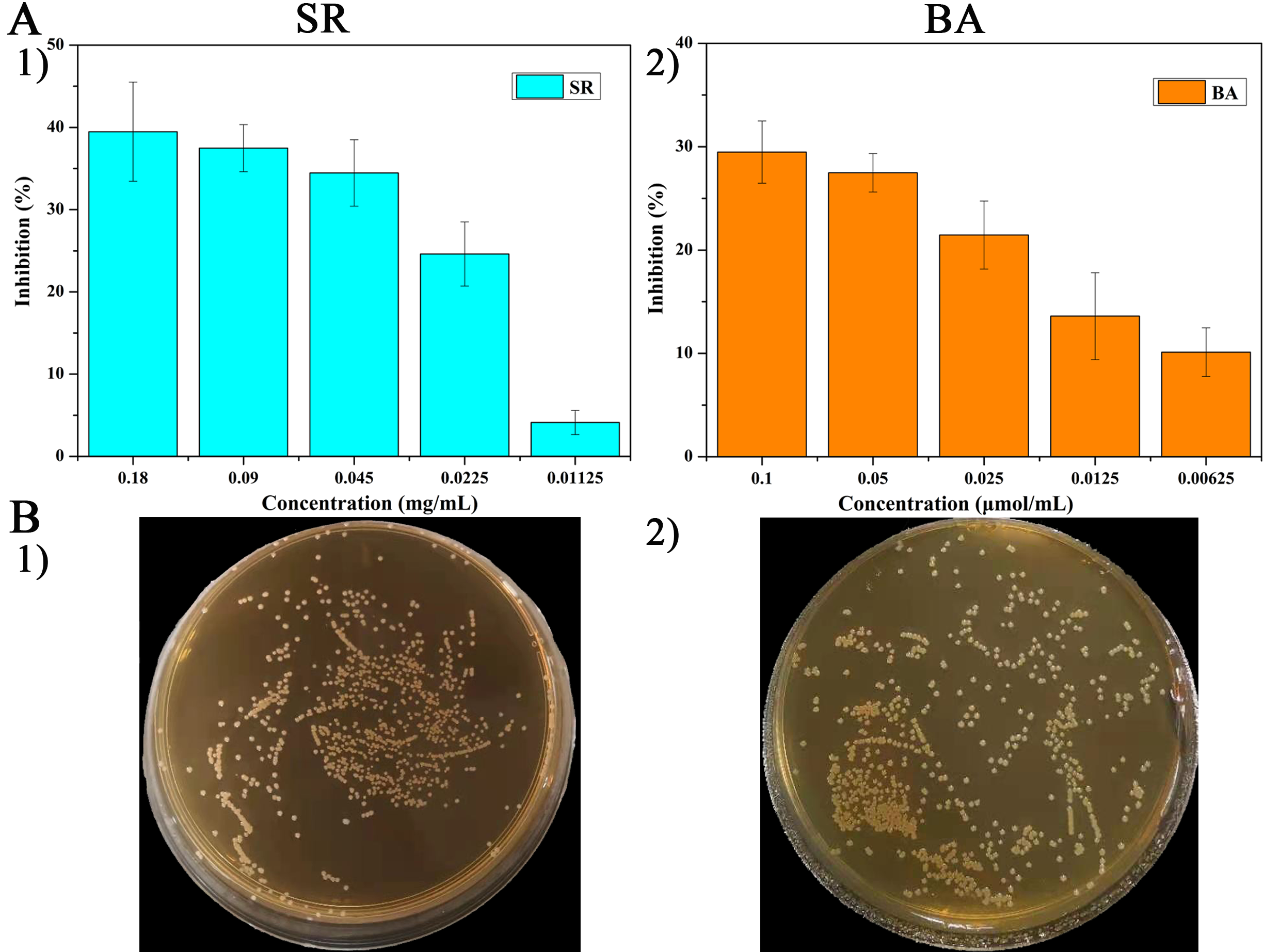


**Fig. S4** Bacteriostatic activity; (a) Inhibition rate of SR and BA at different concentrations against *S.aureus*; (b) Bacterial colonies on culture medium treated with SR and BA.


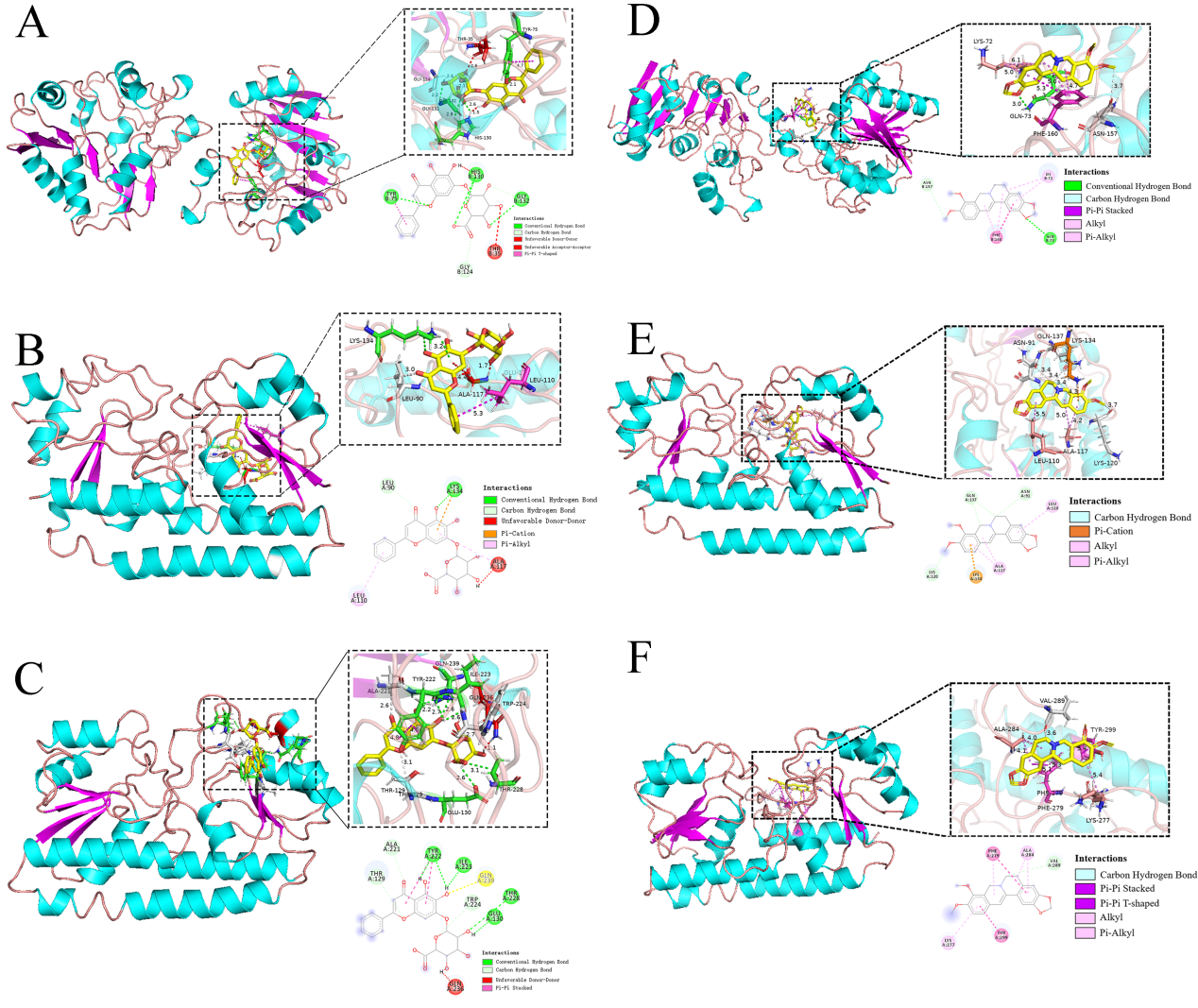


**Fig. S5** Molecular docking study. A. Baicalin with *nagB* test; B. Baicalin with *mntC* test; C. Baicalin with *mntA* test; D. Berberine with *nagB* test; E. Berberine with *mntC* test; F. Berberine with *mntA* test.

The visual analysis results of the mode with the highest molecular docking score were shown in Fig. S5. The dashed line on the way represented the interaction, whose value was the bond length, and the type of interaction was distinguished by different color phases. Baicalin interacted with four amino acids in *nagB*, and its action types included: conventional hydrogen bond, carbon hydrogen bond and π-π stacking (Fig. S5A). The type of interaction between baicalin and amino acids on *mntA* was similar (Fig. S5C). Baicalin also formed π-cation and π-alkyl interaction with amino acids in *mntC* (Fig. S5B). Similarly, the interaction force between amino acids on *mntA*, *mntC* and *nagB* and berberine were mainly carbon hydrogen bond, π-cation, π-alkyl, π-π stackeing, π-π T-shaping, alkyl (Fig. S5D-F). In conclusion, baicalin and berberine could bind amino acid fragments on *nagB*, *mntA* and *mntC* with high affinity through weak bonding. It also affected the amino sugar decomposition and peptidoglycan biosynthesis of bacteria. In addition, the manganese ion transporter was downregulated to reduce the immune escape and attributed to its well antibacterial activity.

**Table S1** Binding energy in molecular bocking

| Ligand | Receptor | Affinity (kcal/mol) |
| --- | --- | --- |
| Berberine | nagB | -8.5 |
|  | mntA | -7.9 |
|  | mntC | -7.9 |
| Baicalin | nagB | -9.3 |
|  | mntA | -7.9 |
|  | mntC | -8.7 |
